# Supplementary material for: How animal ethics committees make decisions – a scoping review of empirical studies
Source: PLoS One. 2025 Mar 17;20(3):e0318570. doi: 10.1371/journal.pone.0318570 (PMC11913294; doi:10.1371/journal.pone.0318570)
Supplement: S2 — (DOCX) [file pone.0318570.s002.docx]

# Search Strategy for Scoping review

## Pubmed

(("animal welfare bodies"[tiab] OR "ECAE" OR "ethics committee for animal experimentation" OR "animal ethics committee*"[tiab] OR "animal care committee*"[tiab] OR "animal welfare body"[tiab] OR "animal welfare committee*"[tiab] OR "animal use committee*"[tiab] OR "institutional animal care" OR "animal ethical review body" OR "committee for the protection of animals"[tiab] OR "animal experimentation oversight committee"[tiab] OR "committee on animal research"[tiab] OR "animal ethics management committee"[tiab] OR "animal ethics council"[tiab] OR "ethics committee for animal experimentation"[tiab] OR "council on animal care"[tiab] OR "Institutional Animal Care and Use Committee*"[tiab] OR"AWERB" OR "IACUC"[tiab] OR"Animal Care Committees"[Mesh] OR (("Ethics Committees"[Mesh:NoExp] OR "Ethics Committees, Research"[Mesh:NoExp]) AND ("Animal Experimentation"[Mesh:noexp] OR "Animal Use Alternatives"[Mesh])) AND ("Decision Making"[Mesh] OR "decision-making" OR "decision process*" OR "decision procedure*" OR "overs*"[tiab] OR "advi*"[tiab] OR "deliberation process*" OR "ethical evaluation" OR "assessment" OR "choice behav*" OR "animal use protocol" OR "ethical review"[Mesh])) AND (2012/1/1:2024/6/3[pdat])) NOT (("animal welfare bodies"[tiab] OR "ECAE" OR "ethics committee for animal experimentation" OR "animal ethics committee*"[tiab] OR "animal care committee*"[tiab] OR "animal welfare body"[tiab] OR "animal welfare committee*"[tiab] OR "animal use committee*"[tiab] OR "institutional animal care" OR "animal ethical review body" OR "committee for the protection of animals"[tiab] OR "animal experimentation oversight committee"[tiab] OR "committee on animal research"[tiab] OR "animal ethics management committee"[tiab] OR "animal ethics council"[tiab] OR "ethics committee for animal experimentation"[tiab] OR "council on animal care"[tiab] OR "Institutional Animal Care and Use Committee*"[tiab] OR"AWERB" OR "IACUC"[tiab] OR"Animal Care Committees"[Mesh] OR (("Ethics Committees"[Mesh:NoExp] OR "Ethics Committees, Research"[Mesh:NoExp]) AND ("Animal Experimentation"[Mesh:noexp] OR "Animal Use Alternatives"[Mesh])) AND ("Decision Making"[Mesh] OR "decision-making" OR "decision process*" OR "decision procedure*" OR "overs*"[tiab] OR "advi*"[tiab] OR "deliberation process*" OR "ethical evaluation" OR "assessment" OR "choice behav*" OR "animal use protocol" OR "ethical review"[Mesh])) AND (2012/1/1:2023/6/1[pdat]))

## Scopus

(TITLE-ABS-KEY("animal welfare bodies" OR "ECAE" OR "ethics committee for animal experimentation" OR "animal ethics committee*" OR "animal care committee*" OR "animal welfare body" OR "animal welfare committee*" OR "animal use committee*" OR "institutional animal care" OR "animal ethical review body" OR "committee for the protection of animals" OR "animal experimentation oversight committee" OR "committee on animal research" OR "animal ethics management committee" OR "animal ethics council" OR "ethics committee for animal experimentation" OR "council on animal care" OR "Institutional Animal Care and Use Committee*" OR AWERB OR IACUC OR "Animal Care Committees" OR (("Ethics Committees" OR "Ethics Committees, Research") AND ("Animal Experimentation" OR "Animal Use Alternatives"))) AND TITLE-ABS-KEY("Decision Making" OR decision-making OR "decision process*" OR "decision procedure*" OR overs* OR advi* OR "deliberation process*" OR "ethical evaluation" OR assessment OR "choice behav*" OR "animal use protocol" OR "ethical review")) AND ( LIMIT-TO ( PUBYEAR,2023) OR LIMIT-TO ( PUBYEAR,2022) OR LIMIT-TO ( PUBYEAR,2021) OR LIMIT-TO ( PUBYEAR,2020) OR LIMIT-TO ( PUBYEAR,2019) OR LIMIT-TO ( PUBYEAR,2018) OR LIMIT-TO ( PUBYEAR,2017) OR LIMIT-TO ( PUBYEAR,2016) OR LIMIT-TO ( PUBYEAR,2014) OR LIMIT-TO ( PUBYEAR,2015) OR LIMIT-TO ( PUBYEAR,2013) OR LIMIT-TO ( PUBYEAR,2012) )

## Web of science (core collection)

(TS=(("Decision Making" OR decision-making OR "decision process*" OR "decision procedure*" OR “overs”* OR “advi*” OR "deliberation process*" OR "ethical evaluation" OR “assessment” OR "choice behav*" OR "animal use protocol" OR "ethical review"))) AND TS=(("animal welfare bodies" OR “ECAE” OR "ethics committee for animal experimentation" OR "animal ethics committee*" OR "animal care committee*" OR "animal welfare body" OR "animal welfare committee*" OR "animal use committee*" OR "institutional animal care" OR "animal ethical review body" OR "committee for the protection of animals" OR "animal experimentation oversight committee" OR "committee on animal research" OR "animal ethics management committee" OR "animal ethics council" OR "ethics committee for animal experimentation" OR "council on animal care" OR "Institutional Animal Care and Use Committee*" OR "awere" OR "IACUC" OR "Animal Care Committees" OR (("Ethics Committees" OR "Ethics Committees, Research") AND ("Animal Experimentation" OR "Animal Use Alternatives")) )) and 2023-06-01/2024-06-03 (Publication Date)
