# Supplementary material for: How animal ethics committees make decisions – a scoping review of empirical studies
Source: PLoS One. 2025 Mar 17;20(3):e0318570. doi: 10.1371/journal.pone.0318570 (PMC11913294; doi:10.1371/journal.pone.0318570)
Supplement: Attachment — Submitted filename: rebuttal letter.docx [file pone.0318570.s006.docx]

# Rebuttal letter

We wish to thank both reviewers for their time in carefully reviewing our manuscript. We have responded to each reviewer in turn with comments (in blue). The mentioned changes have been made to the manuscript

## Comments from reviewer 1

Dear Authors, Thank you for revising your manuscript according to the reviewers' comments. Their manuscript is now ready for publication.

Dear Reviewer,

Thank you for your encouraging response to our manuscript.

## Comments from reviewer 2

Thank you for carefully considering and applying my comments into the revised manuscript. The manuscript is much improved. As stated in my previous review, this manuscript covers an important topic and provides very pertinent and well-presented recommendations for animal ethics review committees. I will be excited to see this manuscript published and utilized to improve the processes involved in decision-making by animal ethics review committees.

Dear reviewer,

Thank you for engaging with our manuscript on such a detailed level. We too hope this manuscript is used to improve the decision making process and we truly appreciate the attention to detail and the helpful suggestions to refine our manuscript.

While I feel the authors have adequately addressed, responded to, and incorporated my comments to the previous version, in this version, I noticed a few places where minor revisions are warranted. Please see below:

1. Results line 143 - I believe the number 586 should be updated to 662 to match Figure 1?

Thank you for spotting this issue, the number has been changed to reflect the updated search.

2. Results line 144 - Please spell out "eighty-three" as this number starts a sentence.

This has been changed.

3. Results lines 144-150 - Throughout this sentence, I suggest using a softer word other than "wrong" -- perhaps "inapplicable" or "irrelevant" in place of the word "wrong." Authors - please feel free to use your discretion here, this is just a suggestion!!

Thank you for this suggestion, we have replaced the word “wrong” with the word “irrelevant” and updated the PRISMA diagram to reflect these changes

4. Results line 192 - Should reference 35 be included in the first set of parentheses with references 30 and 38?

Yes, the reference has been brought into the first set or parenthesis

5. Results line 196 - I think the semi-colon is misplaced, instead there should be a comma after the reference list (before the word "while").

Thank you for pointing this out. The changes have been implemented

6. Results line 203 - The word "in" is not needed.

Thank you, it has been removed

7. Results line 244 - Please check references here. Should there be three or four total supporting citations/studies? Also, reference 35 is not included in this list but is referenced in the next sentence, and reference 31 is not cited again at all in this paragraph.

31 has been removed (it is the study we decided to exclude) and 35 has been added to the topic sentence

8. Results line 259 - Missing space between sentences.

Thank you, a space has been added

9. Discussion line 296 - Missing period between sentences.

Thank you, this has now been added

10. Throughout - there are few sentences with double periods at the end (e.g., lines 177, 228, 241).

Thank you for pointing these out. All double periods have been removed and the document has been checked for any more.
