# Supplementary material for: How animal ethics committees make decisions – a scoping review of empirical studies
Source: PLoS One. 2025 Mar 17;20(3):e0318570. doi: 10.1371/journal.pone.0318570 (PMC11913294; doi:10.1371/journal.pone.0318570)
Supplement: Attachment — Submitted filename: Comments.docx [file pone.0318570.s003.docx]

Dear Editor, Dr. Jayonta Bhattacharjee

Thank you for your request dated 22 August 2024 concerning the evaluation of manuscript number PONE-D-24-34445, entitled (How do Animal Ethics Committees make decisions – a scoping review of empirical studies). Bellow my comments on this manuscript:

**General comment**: Well written article, has viable information about topic of this manuscript.

**Title** of this manuscript is excellent. **The abstract** is covered this manuscript completely.

**Introduction:**  readable, comprehensive, and covering the subject quite right. It is consist of a modern and important references.

**Methods**: Clear and complete.

**Results:** good and clear.

**Discussion:** excellent and clear.

**Recommendations and Limitations:** excellent**.**

**Conclusion**: Authors wrote the conclusion of their study in abbreviated. I suggest that they rewrite the conclusion in detail, according to the results of their study.

**References:** excellent, clear, modern and important references.

- Most references without Doi or links.
- Some references are old, such as 1959, 1986 and 1987. I suggest replacing them with modern references.

**Table:** excellent and clear**.**

**Figures:** good and clear.
