# Supplementary material for: How animal ethics committees make decisions – a scoping review of empirical studies
Source: PLoS One. 2025 Mar 17;20(3):e0318570. doi: 10.1371/journal.pone.0318570 (PMC11913294; doi:10.1371/journal.pone.0318570)
Supplement: Attachment — Submitted filename: Response to reviewers.docx [file pone.0318570.s005.docx]

Rebuttal letter

We appreciate the opportunity to resubmit our manuscript to PLOSONE and thank the reviewers for their assiduous attention to detail and helpful suggestions on how to improve the manuscript. The comments are presented below followed by our responses (in blue). The changes have been made to the manuscript and have been tracked for clear identification.

## **Comments from reviewer 1**

Thank you for your request dated 22 August 2024 concerning the evaluation of manuscript number PONE-D-24-34445, entitled (How do Animal Ethics Committees make decisions – a scoping review of empirical studies). Bellow my comments on this manuscript:

**General comment**: Well written article, has viable information about topic of this manuscript.

**Title** of this manuscript is excellent. **The abstract** is covered this manuscript completely.

**Introduction:**  readable, comprehensive, and covering the subject quite right. It is consist of a modern and important references.

**Methods**: Clear and complete.

**Results:** good and clear.

**Discussion:** excellent and clear.

**Recommendations and Limitations:** excellent**.**

**Conclusion**: Authors wrote the conclusion of their study in abbreviated. I suggest that they rewrite the conclusion in detail, according to the results of their study.

**References:** excellent, clear, modern and important references.

- Most references without Doi or links.
- Some references are old, such as 1959, 1986 and 1987. I suggest replacing them with modern references.

**Table:** excellent and clear**.**

**Figures:** good and clear.

RESPONSE: Thank you for your encouraging words and helpful suggestions to improve our paper. We have expanded the conclusion as you have suggested. It now reads:

Ethical review of animal protocols is a complex and contentious process on which animals depend for their protection. Through this review we have created a clearer picture of the decision making process and charted some of the deep seated issues surrounding the decision making process. At each stage of the evaluation, deficits were found. The scientific validity of a protocol is often neglected, the 3R assessment tends to focus on refinement rather than reduction or replacement, and the harm benefit analysis often focusses on harms rather than an in –depth weighing of interests. The committees themselves influenced these processes significantly. Committee composition affects the knowledge and expertise available to address key issues, but also the focus of the review. Problems with group dynamics and independence of committees were also discussed. Remedies to address these issues were suggested and although the recommendations given above may be costly, they reflect the deep responsibility for those involved in animal experimentation to protect the animals they work with and ensure responsible research. Further research should delve into the ethical issues debated and the ethical theories informing decision making along with testing measures to address the aforementioned issues.

RESPONSE to comments about the references:

Thank you for raising this issue. There are some older references that we felt were important to keep, for example, Russel and Birch who first proposed the 3R principle in 1959 or legislation that was introduced in the 70s-80s. Where possible we replaced other literature such as Singer 1987 with more up to date references.

## Comments from reviewer 2 with responses

Overall response; Thank you very much for your careful evaluation of our paper. We truly appreciate your attention to detail and your helpful suggestions to improve our work. Below are responses to the individual issues raised which we hope have been addressed to your satisfaction.

### Major revisions

1. In general, the Results section is too long and can be improved by revising to be more concise. Common themes between articles should be summarized in tables or figures, or using much more concise language in the main text. One suggestion is to move all details about the search and selection process (including Figure 1) to the Methods section, where I believe it fits more appropriately.

RESPONSE: Thank you for this point. We have rewritten the results section to make it more succinct We have kept the PRISMA diagram (figure1) and the details about the selection process in the results section to comply with the PRISMA SCR checklist

2. I am confused about the "one additional paper found through other sources" (lines 141-142). How was this paper found? Why was it not found during the systematic search using described methods? Which paper is this? Throughout the results, it is unclear whether this additional 13th paper is included or not. For example, on lines 154-155, it is stated that "half of the retrieved literature originated in the US..." but half of 13 is 6.5. Please clarify if you mean 6 or 7 papers originated in the US. Moreover, in Figure 1, studies included for review is noted to be n=12. Should this be n=13? Is the method by which this 13th paper was found included in the figure? If not, please include or clarify.

RESPONSE: Thank you for raising this issue, the additional paper was not found systematically but by extended reading around the topic. We have decided to remove references to the additional paper (Jankoski) The results section has been edited to ensure correct numbers are used throughout

3. Methods state that the search was updated later in 2024 (March or June - date format in Methods needs to be clarified, see below); however, Figure 1 is dated 15th January 2024. Were there no changes implemented after the update? Please clarify the discrepancy.

RESPONSE: Thank you for your keen observation, the PRISMA diagram has been updated to reflect the updated search.

4. The way in which Table 1 and Figure 2 are presented makes it difficult for the reader to cross-reference the studies included. Studies are dually numbered, 1-13 for Table 1 and 25-37 for the reference list. I believe the table and figure could be combined to limit confusion and present the themes in conjunction with relevant details about each study If combining proves difficult due to the amount of information, I recommend converting Figure 2 into a table (similar to Table 2 in Budda & Pritt, 2020 - referenced above). Presenting Figure 2 in a more explicit manner may help to reduce text in Results section in general as well (see major revision #1).

RESPONSE: Thank you for raising this point. We have converted figure 2 into a table (table 2) and we have removed the dual numbers, opting instead for including the first author and date. We have then removed redundant sentences containing the same information as the table to make the results section more succinct.

5. In the Results section, included studies are described as presenting "self-reported data or other data collection methods" (line numbers not provided). Please clarify earlier in the manuscript when describing selection criteria. What types of empirical data were you looking for, specifically? I can deduce that you were looking for either self-reported data, or data that were collected from committee documents. However, this is not explicitly clear to the reader. Were there other types of studies considered?

RESPONSE: Thank you for this point. For our search, all types of empirical data related to how committees make decisions were considered. The search strategy did not include the types of data collected so if it was relevant to the research question it was included in the results. We have clarified this part (see line number 121).

6. While I appreciate the inclusion of a "Limitations" section, I believe this section is severely lacking. Please discuss other major limitations in this study such as biases in self-reported data, or the potential for missing information from committee documents that may not have covered the full breadth of discussions that took place.

RESPONSE: Thank you for this point. We have added more detail to the limitation section (see line numbers 381-384)

7. Please incorporate Budda & Pritt (2020) - referenced above - into the Discussion section of this paper. There is some overlap that needs to be discussed and justified. As stated above, I do feel that the objective, content analysis, and conclusions of the submitted paper differ enough from Budda & Pritt, 2020 to justify its publication after major revisions; however, I think the overlap can be addressed to strengthen the conclusions presented here and perhaps eliminate some redundancy to make the submitted paper more concise.

RESPONSE: Thank you for the suggestion. We have added reference to this paper in the introduction section of our paper (see lines 92-95)

### Minor revisions: 1. In the abstract, please define all acronyms (i.e., AECs, IACUCs, 3R).

Definitions have been added

2. Line 43: Please clarify whether "8 million animals" includes all taxa or just vertebrates (as in the second part of the sentence).

The referenced report included all animals as defined by EU law. The scope includes vertebrates and a limited number of non-vertebrates. Given the actual numbers used a small note (mostly vertebrates) was included in the main text.

3. Lines 114-115: Clarify whether dates provided are mm.dd.yyyy or dd.mm.yyyy format.

A small note has been added

4. Line 127: Data should be plural -- "data were collated"

The correction has been made

5. Lines 139 and 142: When starting a sentence with a number, spell out the number.

This has been added

6. Figure 1: References removed for other reasons should be n=0? Current reads "n=".

This figure has been replaced with an updated figure which resolves this issue

7. Provide reference to Table 1 in main text.

Reference to the table has been made

8. Table 1: Formatting of headings needs to be adjusted to be consistent.

This has been adjusted

9. Table 1: Clarify for each study if it includes empirical data from multiple IACUCs/AECs or only one. An added column could provide the number of studies summarized in each paper.

An extra column has been added to the table to clarify this issue

10. Figure 1: Remove extra comma for study #s discussing "Independence of review."

Figure 1 has been removed

11. Figure 1: Please improve consistency of capitalization/sentence case.

Figure 1 has been removed

12. In the Results (line numbers not provided), it is mentioned that methods of reduction included "animal numbers of an experiment and statistical methods rather than any other method of reduction." Please clarify somewhere what other suggested methods of reduction might be.

A short note has been added here to provide an example of a different method of reduction

13. Section c) Assessment of scientific validity - second line (line numbers not provided): Should read "...US where assessment of scientific validity is not required..."

Change has been made

14. Section c) Committee-related factors - first three lines (line numbers not provided): Unclear why these lines are formatted differently (indented and italicized) than the rest of the section.

Formatting has been changed to be consistent

15. Sentence in Results section (no line numbers provided) has incorrect grammar. Currently reads "Variances in perceived ability to influence discussions was reported by committee members in one study (35)." Should read "Variance in perceived ability to influence discussions was reported by committee members in one study (35)." OR "Variances in perceived ability to influence discussions were reported by committee members in one study (35)."

This sentence has been removed in an effort to make the results section more succinct.

16. In Discussion section (no line numbers provided), please clarify what is meant by "animal welfare experts" if different/separate from scientific researchers to clarify claims about scientists' biases toward research.

We included a suggestion of veterinarians here

17. In Recommendation #4 (line numbers not provided), please capitalize the word "Training."

Change has been incorporated
